# Mutational Analysis of the Nsa2 N-Terminus Reveals Its Essential Role in Ribosomal 60S Subunit Assembly

**DOI:** 10.3390/ijms21239108

**Published:** 2020-11-30

**Authors:** Helge Paternoga, Alexander Früh, Ruth Kunze, Bettina Bradatsch, Jochen Baßler, Ed Hurt

**Affiliations:** Biochemie-Zentrum der Universität Heidelberg (BZH), Im Neuenheimer Feld 328, 69120 Heidelberg, Germany; helge.paternoga@googlemail.com (H.P.); al.frueh@outlook.de (A.F.); ruth.kunze@bzh.uni-heidelberg.de (R.K.); bbradatsc@googlemail.com (B.B.); ed.hurt@bzh.uni-heidelberg.de (E.H.)

**Keywords:** ribosome assembly, ribosome biogenesis, Nsa2, Nog1, Rsa4, TINP1, Rea1, Mdn1

## Abstract

The ribosome assembly factor Nsa2 is part of the Rea1-Rsa4-Nsa2 interconnected relay on nuclear pre-60S particles that is essential for 60S ribosome biogenesis. Cryo-EM structures depict Nsa2 docked via its C-terminal β-barrel domain to nuclear pre-60S particles, whereas the extended N-terminus, consisting of three α-helical segments, meanders between various 25S rRNA helices with the extreme N-terminus in close vicinity to the Nog1 GTPase center. Here, we tested whether this unappreciated proximity between Nsa2 and Nog1 is of functional importance. Our findings demonstrate that a conservative mutation, Nsa2 Q3N, abolished cell growth and impaired 60S biogenesis. Subsequent genetic and biochemical analyses verified that the Nsa2 N-terminus is required to target Nsa2 to early pre-60S particles. However, overexpression of the Nsa2 N-terminus abolished cytoplasmic recycling of the Nog1 GTPase, and both Nog1 and the Nsa2-N (1-58) construct, but not the respective Nsa2-N (1-58) Q3N mutant, were found arrested on late cytoplasmic pre-60S particles. These findings point to specific roles of the different Nsa2 domains for 60S ribosome biogenesis.

## 1. Introduction

The assembly of eukaryotic ribosomes starts in the nucleolus with the transcription of the pre-rRNA by RNA polymerases I and III. Throughout the subsequent maturation steps, the pre-rRNA is decorated stepwise with biogenesis factors and ribosomal proteins. During this process, the nascent pre-ribosomal particles are transported from the nucleolus into the nucleoplasm, before the final export into the cytoplasm. Along this pathway, the pre-rRNA is modified with the help of snoRNAs, processed by endo- and exonucleases and folded by specific biogenesis factors. In this complicated process, a set of ca. 200 ribosome biogenesis factors (also called assembly factors) transiently interact with the developing pre-ribosomal particles (for recent reviews, see [1,2,3]).

The assembly pathway for the 60S subunit emerges after cleavage of the common 35S pre-rRNA inside the ITS1 region and involves several structural hallmarks. At first, rRNA folding within the pre-60S particle is obtained via a ring-like intermediate, where the 5′ and 3′ ends of the 27S rRNA assemble together first and then integrate stepwise the intermediate rRNA sequences into the nascent pre-60S ribosome [4,5,6]. A next characteristic in 60S assembly is the removal of the so-called foot structure, which includes the ITS2 rRNA sequence, by the Las1 subcomplex and the RNA exosome [7,8,9,10,11]. During 60S assembly, also integration of the 5S RNP occurs, which subsequently has to undergo a rotation to reach its mature position [5,12,13,14]. It is discussed that the 5S rRNP rotation is coupled to a mechano-chemical force generated by the Rea1 AAA-ATPase that is transmitted via the Rix1 subcomplex and Rsa4-Nsa2 dimer into the pre-ribosome [15,16,17], but the exact mechanism of this process remains elusive. This rearrangement, however, is strictly coupled to the release of Rsa4, the Rix1 subcomplex [17] and the Nog2 GTPase, which allows the construction of the L1 stalk and the recruitment of the Nmd3 export adaptor [18,19,20,21,22,23]. Despite the strong interaction of Rsa4 and Nsa2, Nsa2 stays associated at the still immature catalytic center (peptidyl transferase center, PTC) of the derived 60S pre-ribosomal particle [16,17,22] and is later released by a yet unknown mechanism. Nsa2 is functionally linked to the Nog1 GTPase and both bind to the nascent PTC at an early nucleolar stage [4] and their release appears to be an important trigger for PTC maturation, including the rearrangement of the rRNA helix H89. Nsa2 is believed to be released prior to nuclear export of the pre-60S particle, as the Nsa2 C-terminus blocks recruitment of Nmd3 [22]. The Nog1 GTPase is released in a stepwise manner: first, the N-domain and the GTPase domain appear to be released by a GTP-dependent mechanism [22,24], whereas the meandering C-terminus that inserts into the nascent exit tunnel is released in a Drg1-dependent manner, together with the assembly factor Rlp24 [25,26]. The following final maturation steps include the cytoplasmic recycling of biogenesis factors amongst them Nmd3 [27], Tif6 [28], Mrt4 [29,30,31], which finally results in mature 60S subunits.

Whereas most steps in maturation of the PTC could meanwhile be structurally analyzed by cryo-EM, the mechanisms that drive these dynamic binding and release reactions are only poorly understood. In particular, the role of the extremely conserved ribosome assembly factor Nsa2, the ortholog of human TINP1 [32], is still rather unclear. Nsa2′s function appears to be tightly coupled with pre-rRNA rearrangement to obtain the functional catalytic center of the 60S subunit [16]. Interestingly, it has been observed that TINP1 overexpression promotes proliferation of tumor cells [33], which is consistent with the intimidate link of ribosome biogenesis to cancer development via the p53 pathway (for review, see [1,34,35]).

**Figure 1:**
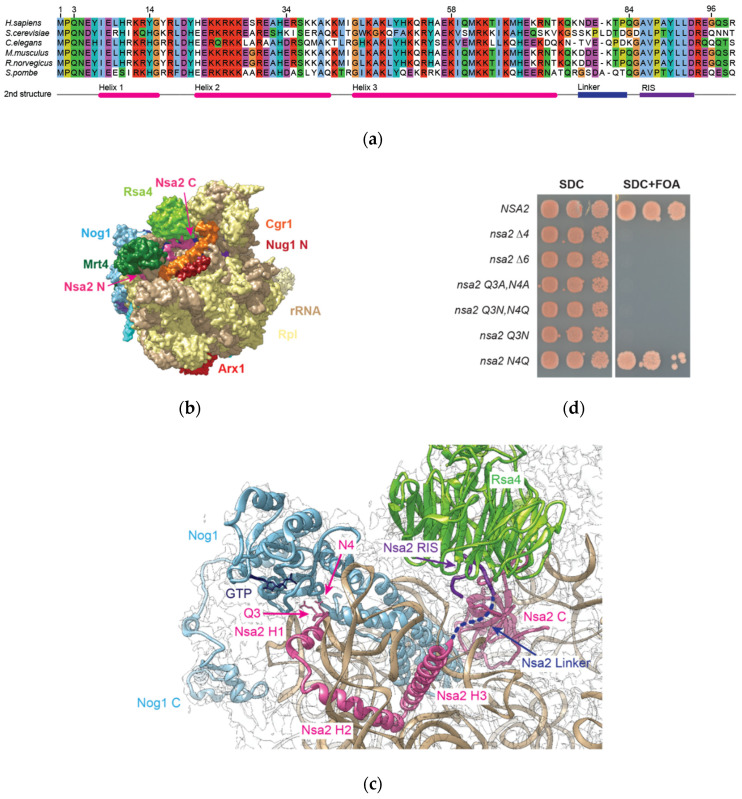
Structure and position of Nsa2 inside a pre-60S ribosome. (**a**) Sequence alignment of different Nsa2 homologs from the indicated organisms, using Clustal Omega [37] and Jalview [38]. The indicated secondary structure below the alignment is based on the cryo-EM structure (pdb: 3jct, chain r) [36], with labeling of the N-terminal α-helical segments Nsa2 Helix1-3, the flexible internal linker and the Rsa4 interacting sequence (RIS). (**b**) Cryo-EM structure of the Nog2-derived pre-60S particle (EMD: 6615; pdb: 3jct), illustrating the position of associated biogenesis factors by using the Chimera software [39] (**c**) Zoom of the Nog2-pre-60S cryo-EM structure, showing the proximity between Nsa2 N-terminal extension and the Nog1 GTPase domain including the position of the Nog1 bound GTP. Furthermore, the Nsa2 RIS and linker is shown in purple and blue, respectively, whereas the remaining amino acids of Nsa2 are depicted in pink. The Nog1 GTPase is shown in light blue, bound GTP in dark blue, and Rsa4 in green [36]. (**d**) Growth analysis of N-terminal Nsa2 truncations. The *nsa2* shuffle strain (nsa2∆) was transformed with the plasmid pMT Leu2 P_NSA2_ *NSA2* coding for the indicated *NSA2* alleles. The transformants were incubated on SDC-Leu and SDC+FOA at 30 °C for four days.

Nsa2 is known to be a component of the Rea1 ATPase-Rsa4-Nsa2 array that is indispensable for 60S assembly in eukaryotes [16]. The protein has been detected in cryo-EM structures of nucleolar and nuclear particles that cover its recruitment via its Rsa4 bound state in a Rix1-purified particle until a nuclear Rsa4 released state [4,22,36]. Apparently, during all these maturation stages, Nsa2 does not significantly change its conformation and location on these evolving pre-60S particles. In particular, the meandering Nsa2 N-terminal extension contacts various RNA elements of the 25S rRNA, including rRNA helices H89, H42, H43, H44, H97, and H91 (see Figure 1c).

In our current study, we discovered an essential amino acid at the tip of the Nsa2 N-terminus (Nsa2 Q3) that is adjacent to the GTPase domain of Nog1, as revealed in recent structural pre-60S models. To further shine light on the key role of this residue for 60S assembly, we performed a functional characterization of the Nsa2 N-terminal domain, carrying this crucial Q3 residue. This analysis showed the importance of the Nsa2 N-terminus in pre-60S targeting, but its overexpression abolished the release and re-import of the Nog1 GTPase into the nucleus.

## 2. Results

### 2.1. Nsa2 Q3 Residue Contacting the Catalytic Center of the Nog1 GTPase is Crucial for 60S Biogenesis

An assessment of recent structural models derived from cryo-EM structures of various pre-60S particles suggested that the highly conserved Nsa2 N-terminal extension contacts the GTPase domain of Nog1 (Figure 1a–c). Based on the findings that *NSA2* acts as a high-copy suppressor of *nog1* ts mutants [32], and that *nsa2* and *nog1* mutants are synthetically lethal [16], we wanted to understand whether this so far unappreciated proximity between the Nsa2 N-terminal end and Nog1 is of functional importance. For this purpose, we analyzed short N-terminal truncations *(nsa2∆6* or *nsa2∆4)*, which to our surprise already showed a lethal phenotype (Figure 1d, Supplementary Materials Table S1 summarizes the phenotype of mutants analyzed in this study). Notably, the conserved residues at the N-terminal end of Nsa2, in particular Q3 and N4, are oriented towards the Nog1 GTPase domain (Figure 1c). We therefore mutated these residues to alanine (Nsa2 Q3A,N4A) which again resulted in a lethal phenotype (Figure 1d). Unexpectedly, even the conservative double mutation, Nsa2 Q3N,N4Q, changing only the amino acid side chain length by one CH_2_ group, was not able to complement the *nsa2∆* null mutant (Figure 1d). By separately changing Q3 and N4, it turned out that the single Nsa2 Q3N, but not the N4Q mutation, is responsible for the lethal phenotype (Figure 1d). However, the single Nsa2 Q3N mutant strain occasionally showed formation of very small colonies on plates after one week of incubation (data not shown). Therefore, we continued to analyze the completely lethal Nsa2 Q3N,N4Q double mutant in the following.

**Figure 2:**
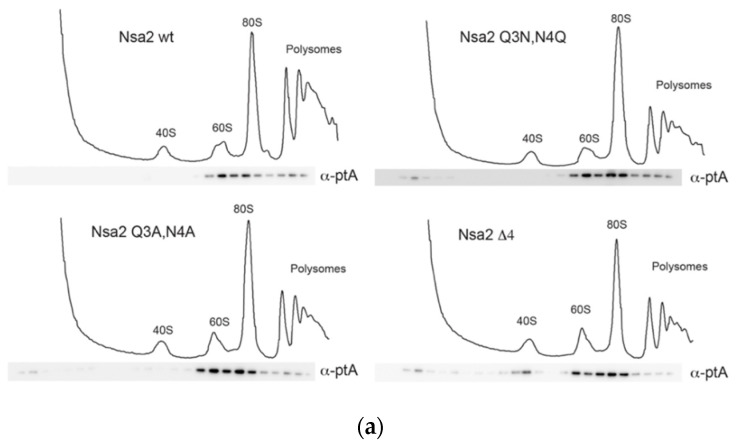

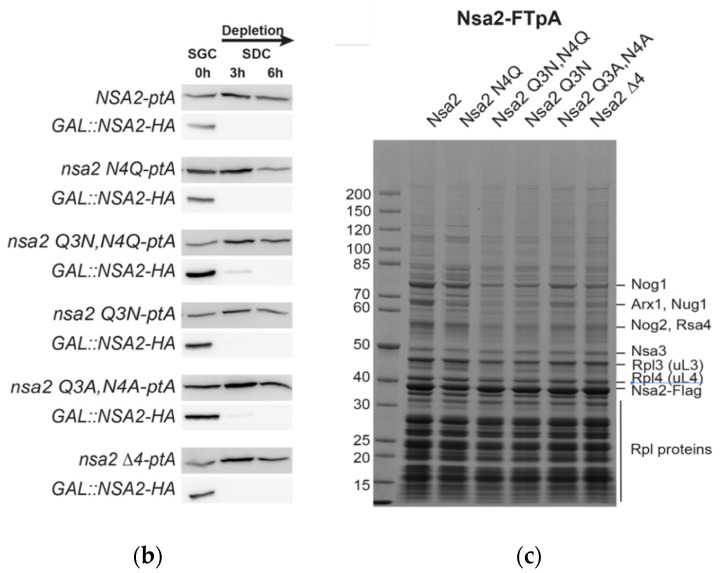
Association of N-terminal Nsa2 mutants with ribosomal particles. (**a**) Whole cell lysate supernatant derived from the Nsa2 shuffle strain (containing pRS316 *NSA2*) expressing the indicated Nsa2-FTpA fusion proteins from a plasmid pMT-Leu2 P_NSA2_ NSA2-L-FTpA was loaded on a 10–50% sucrose gradient. After ultracentrifugation, the gradient was fractionated and the UV profile at 254 nm was recorded (upper profile). Fractions were analyzed by western blotting using an anti-ProtA antibody (lower panel). (**b**,**c**) Isolation of pre-60S particles via N-terminally ProtA-tagged Nsa2 wild type and mutant proteins from a *nsa2∆* depleted background. The *nsa2∆* null strain carrying plasmid YCplac22 *GAL1::NSA2*-L-HA (*GAL::NSA2-HA)* for *NSA2* repression and pMT-Leu2-*NSA2*-L-FTpA (*NSA2-pA*) carrying the indicated *NSA2* wild type and *nsa2* mutant alleles was grown at 30 °C in SGC-Trp-Leu. In order to deplete the Nsa2-HA wild type, cells were shifted to SDC-Trp-Leu for 6 h before purification of the Nsa2 mutant proteins to repress the expression of *GAL::NSA2-HA* (see Materials and Methods). (**b**) Western blot analysis using anti-ProtA and anti-HA antibodies of whole cell lysates to monitor the efficiency of the Nsa2-HA depletion. (**c**) Affinity-purification of pre-60S particles using the indicated Nsa2 bait proteins from a *NSA2* wild type background, which were subsequently analyzed by SDS-PAGE and Coomassie staining. Major co-purifying bands are indicated.

To investigate, whether the lethal phenotype of these *nsa2* mutants (Figure 1d) is due to a defective association with pre-ribosomal particles, we performed sucrose gradient centrifugation combined with western blot analysis. This revealed that the FTpA-tagged Nsa2 proteins were still associated with pre-ribosomal particles (Figure 2a), even in the presence of a Nsa2 wild type copy. In addition, we affinity-purified pre-ribosomal particles from yeast cells, which constitutively expressed the different Nsa2 mutant proteins in *GAL::NSA2* depleted cells (Figure 2b) (see also Materials and Methods). However, these co-precipitated pre-60S particles were not significantly altered when compared to normal wild type pre-60S particles, exhibiting the typical set of pre-60S factors (Figure 2c). Similar results were observed, when another pre-60S factor Arx1 was affinity-purified from these *nsa2* mutant cells (J.B. and B.B., unpublished results). Thus, deletion or mutation of the Nsa2 N-terminal end caused a lethal phenotype, but did not significantly alter the composition Nsa2-associated pre-60S particles.

### 2.2. Overexpression of the Nsa2 N-Terminus Blocks 60S Biogenesis

**Figure 3:**
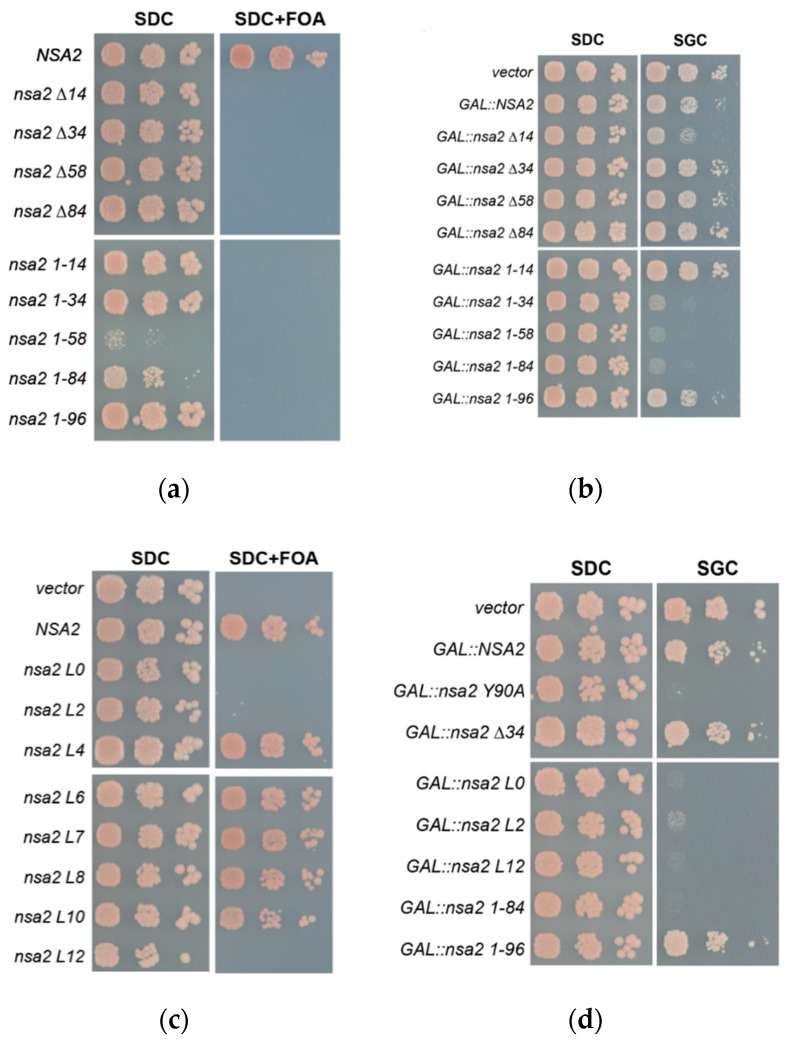
Genetic analysis of Nsa2 N-terminal fragments. (**a**,**c**) The nsa2 shuffle strain (*nsa2*∆) was transformed with the low-copy plasmid YCplac22 (*URA3*) *NSA2*-L-HA expressing the indicated *NSA2* constructs under control of the endogenous *NSA2* promotor. Derived transformants were spotted in 10× dilution series on SDC-Trp and SDC+all+FOA and incubated for 3 days at 30 °C. (**b**,**d**) Wild type strain W303 was transformed with the indicated low-copy plasmid YCplac22 *GAL1::NSA2-HA (GAL::NSA2)* (**b**) or high-copy plasmid YEplac112 *GAL1::NSA2*-Flag (**d**) expressing the indicated *NSA2* alleles under control of the inducible *GAL1* promotor. In the *nsa2* linker mutants (**c**,**d**) the wild type sequence SKPLDTD was replaced with the indicated length of an artificial linker (L) consisting of alternating glycine and serine residues (e.g., L7: GSGSGSG). Transformants were grown at 30 °C for 3 days on SDC-Trp and SGC-Trp plates.

Next, we sought to extend this analysis considering the entire N-terminal domain (Figure 1a). Accordingly, various Nsa2 N-terminal truncations or N-terminal fragments truncated from the carboxy-terminus were expressed and tested for viability in the *nsa2∆* shuffle strain or for a dominant-lethal phenotype in the wild type W303 yeast strain (Figure 3a,b). As anticipated, the N-terminal deletions and the various N-terminal fragments were not able to complement the *nsa2∆* null mutant. However, cells expressing Nsa2-N (1-58) or Nsa2-N (1-84) showed a slow-growth (dominant) phenotype even in the presence of wild type *NSA2* (Figure 3a). To further verify this dominant negative phenotype, we overexpressed the corresponding proteins by the use of the inducible *GAL* promotor in a wild type yeast strain (Figure 3b). In this case, we observed a strong slow-growth phenotype of the *GAL::NSA2-N* (1-34), *GAL::NSA2-N* (1-58) and *GAL::NSA2-N* (1-84). Hence, we also tested whether the Nsa2 Q3,N4 mutants in the full-length Nsa2 protein show a dominant phenotype. However, to our surprise neither the Nsa2 Q3A,N4A nor the Nsa2 Q3N,N4Q mutant induced a dominant-negative growth nor a ribosome assembly defect, as suggested from the cell growth and the ribosome profile analysis (Supplementary Materials, Figure S1a,b).

### 2.3. The Linker between Nsa2 N-Terminus and the Rsa4 Interaction Sequence is Critical for 60S Assembly

Previously, we observed a dominant negative-phenotype for another *nsa2* mutant, Nsa2 Y90A, that was impaired in Rsa4 binding [16] (Figure 3d). Interestingly, in our new study only Nsa2 N-terminal fragments without a Rsa4 interaction sequence (RIS) that harbors the crucial Y90 residue showed a dominant-negative phenotype, whereas Nsa2-N (1-96) that includes the RIS did not (Figure 3b). This finding suggested that the functional connection between the RIS-containing Nsa2 N-terminus and Rsa4 is critical for the overall function of Nsa2. In contrast, the linker region between the N-terminus and the Rsa4 interaction sequence is only weakly conserved. This linker sequence is flexible, as it cannot be seen in cryo-EM structures [4,36]. However, its role could be to generate an optimal spacer/connecter between the RIS and the N-terminally located α-helical segments, in this way also allowing the N-terminal end of Nsa2 to reach the Nog1 GTPase (Figure 1a,c). To test this hypothesis, we replaced the linker sequence (residues 77–83) by an artificial linker (alternating glycine and serine residues), allowing us to also vary the linker length (Figure 3c). When the wild type sequence SKPLDTD was replaced with an artificial linker of the same length (L7: GSGSGSG), the resulting full-length Nsa2 variant was able to complement the *nsa2*∆ null strain. Notably, even longer or short linkers (L4–L10) were tolerated, but if the connecting sequence was ≥12 aa (L12) or ≤2 aa (L2), cells were no longer viable. In addition, the lethal Nsa2 L2 and Nsa2 L12 linker constructs showed a dominant-negative phenotype, when overexpressed in a wild type strain (Figure 3d). Taken together, the N-terminal half of Nsa2 is essential and requires an appropriate connection via a linker sequence to the RIS motif, so that Nsa2-N remains physically connected to the β-propeller domain of Rsa4, which is essential for pre-60S assembly.

### 2.4. The Nsa2 N-Terminus is Responsible for its Recruitment to Pre-Ribosomes

**Figure 4:**
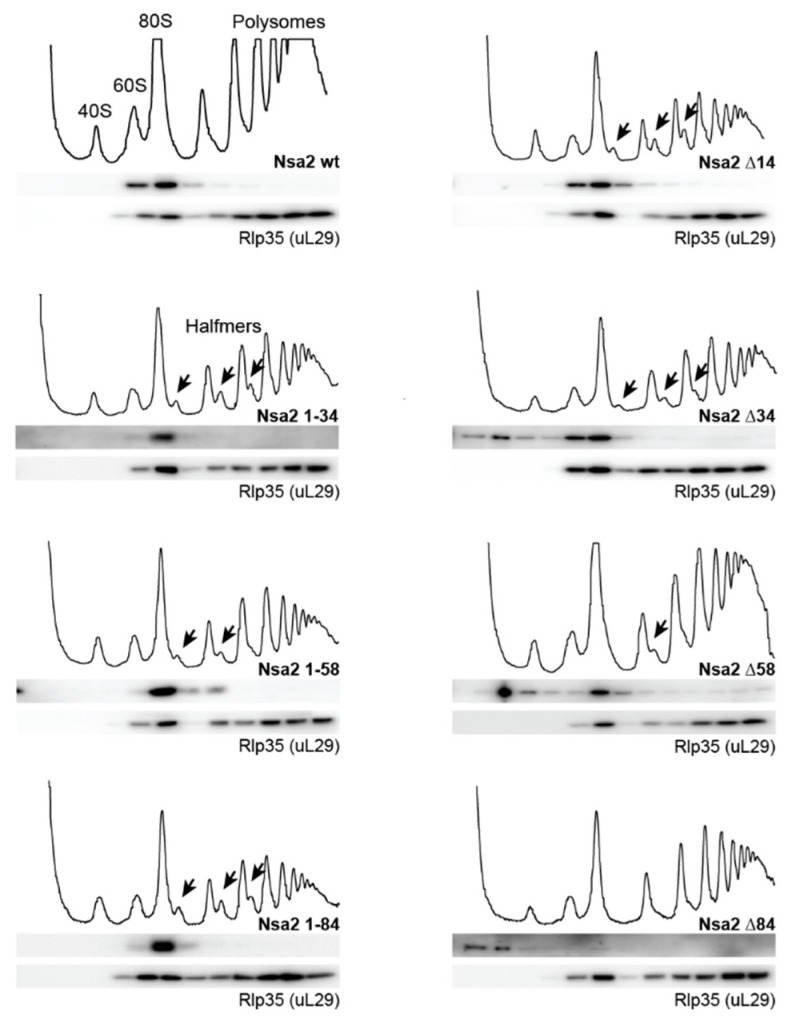
Association of the Nsa2 constructs with pre-ribosomal particles and generation of a half-mer phenotype. Wild type yeast strain W303 was transformed with the indicated *nsa2* alleles inserted into a single-copy plasmid YCplac22 *NSA2*-L-HA under control of the endogenous *NSA2* promotor. Transformants were grown at 30 °C in selective medium SDC-Trp and lysed. Whole cell lysate supernatants were applied on a 10–50% sucrose gradient, centrifuged and fractionated. The rRNA absorption profile was recorded at 254 nm wave length and a western blot analysis using anti-HA antibodies to detect the Nsa2 fragments and an anti-Rpl35 (uL29) antibody to reveal the ribosome distribution, is shown below the profile. Position of 40S, 60S, 80S, and polysomes are indicated. The appearance of half-mers is marked by black arrows.

A dominant-negative phenotype of ribosome biogenesis mutants can be frequently observed when the mutant protein competes with the wild type protein for its binding site at the pre-ribosome and cannot efficiently be released (e.g., Rsa4 E114D [17] or Ytm1 E80A [40]), resulting in a block of downstream maturation steps. To test whether this is also true for the dominant-negative Nsa2 N-terminal fragments, we analyzed their association with 60S pre-ribosomes by sucrose gradient centrifugation and subsequent western blotting (Figure 4). Consistent with the dominant-negative effect, co-expression of the Nsa2 N-terminal fragments caused the appearance of half-mer polysomes (Figure 4, black arrows) that are characteristic for a 60S biogenesis defect. The western blot analysis, detecting the HA-tagged Nsa2 variants, showed that the various N-terminal fragments are sufficient to associate with pre-ribosomal particles. On the other hand, the more amino acids were deleted from the Nsa2 N-terminus, association with pre-60S particles was progressively diminished. As an example, the Nsa2 ∆84 construct nearly lost its binding to pre-ribosomal particles and accordingly did not show a half-mer phenotype in the polysomal profile. In contrast to the ribosomal protein Rpl35 (uL29), no Nsa2 fragment could be found in the polysomes fractions, which contain translational active mature subunits. Therefore, we conclude that the Nsa2 N-terminus is essential for efficiently targeting Nsa2 to pre-ribosomal particles, which can well explain the dominant-negative effect upon overexpression. A recent cryo-EM analysis of early, nucleolar pre-60S particles [4] has depicted the structure of the Nsa2 N-terminus, whereas no obvious EM density for its C-terminal domain could be observed (pdb:6em1, state C). Thus, we conclude that Nsa2 binds via its N-terminus to pre-ribosomal particles and that in a second step its C-terminal domain is stably attached to pre-60S particles during the compaction of the 27S rRNA, whereas Rsa4 is recruited to pre-60S particles at a later time point [4,17].

**Figure 5:**
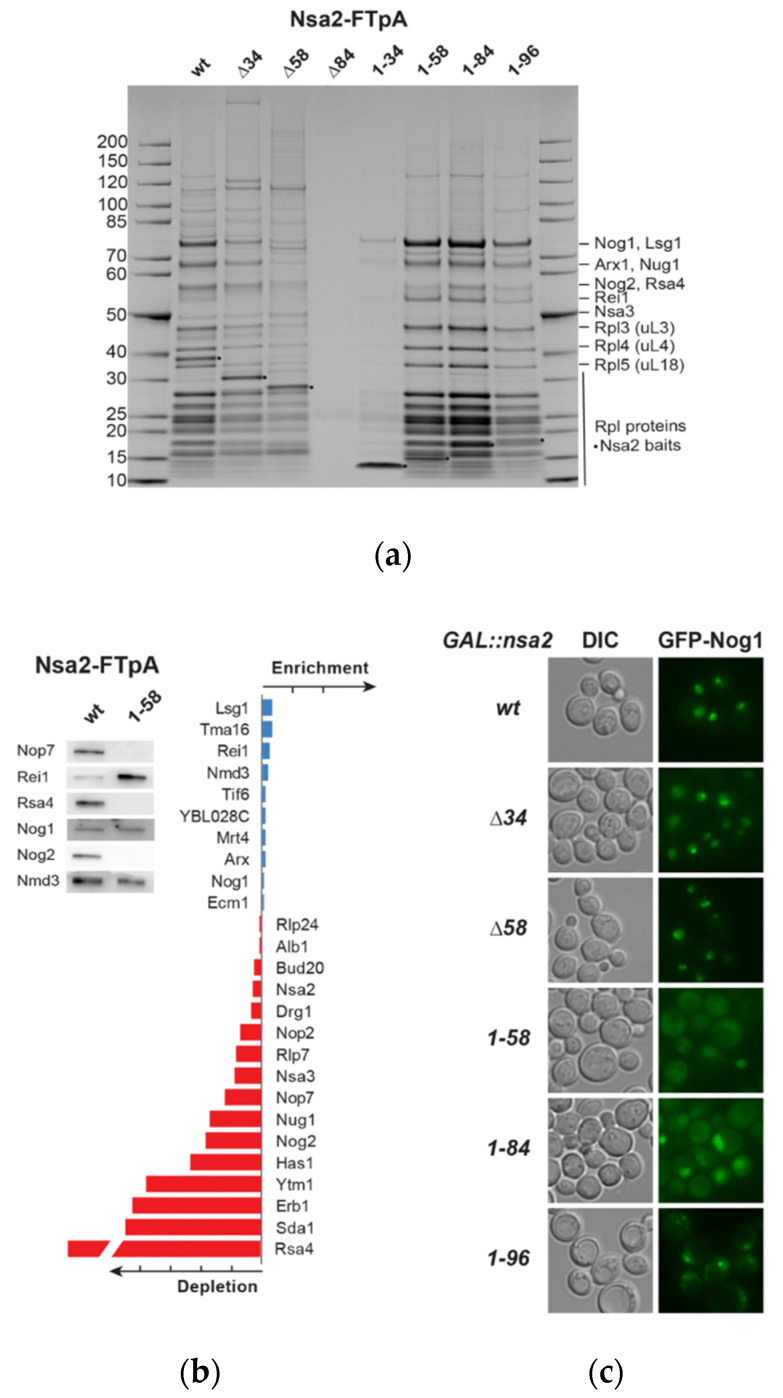
The Nsa2 N-terminal domain binds to pre-60S particles. (**a**) Two-step affinity-purification was done from yeast cells expressing the indicated Nsa2 constructs from YCplac111 *NSA2* L-FTpA plasmids in a wild type background. Final eluates were analyzed by 4–12% gradient SDS-PAGE and Coomassie staining. Labeled biogenesis factor bands were identified by mass spectroscopy. **(b**) Analysis of pre-60S particles affinity-purified by Nsa2 wild type or Nsa2-N (1-58) constructs followed by western blot analysis and semi-quantitative mass spectroscopy analysis using MaxQuant. The iBAQ values of selected biogenesis factors were normalized for the ribosomal protein Rpl4 (uL4) and the ratio of wild type Nsa2 versus Nsa2-N (1-58) (or reciprocal values for depleted factors) are shown. One bar on the horizontal axis corresponds to a 20-fold change either in increase or decrease (see Supplementary Materials Table S2 for complete analysis by the MaxQuant software). (**c**) Subcellular localization of GFP-Nog1 was determined by fluorescence microscopy of yeast cells *GAL1*-promoter induced overexpressing the indicated Nsa2 constructs (YCplac22 *GAL1::NSA2*-L-HA). Overexpression was induced for 6 h by incubation in galactose containing medium (SGC-Trp+2xAde).

To find out at which step ribosome biogenesis is disturbed, we analyzed which pre-60S factors are associated with the Nsa2 N-terminal constructs. Accordingly, Nsa2 N-terminal fragments tagged with Flag-TEV-ProtA (FTpA) were affinity-purified. Most of these Nsa2 N-terminal constructs were found to be associated with pre-60S particles (Figure 5a), except Nsa2-N (1-34), which might be too short to allow a stable interaction as it purified only traces of pre-ribosomal particles (please note that the gradient sedimentation has been done with Nsa2-HA constructs, whereas the purification was done with Nsa2-FTpA fusion proteins). Comparable to the Nsa2 Q3N,N4Q mutant, short N-terminal truncations did not abolish binding to pre-60S particles. However, Nsa2 wild type, nsa2∆34, and nsa2∆58 tend to contain earlier pre-60S biogenesis factors than the Nsa2 N-terminal constructs. To verify this, we performed a western blot analysis and a semi-quantitative mass spectroscopy to compare Nsa2 wild type and Nsa2-N (1-58) associated particles. Both analyses (Figure 5b, Supplementary Materials Table S2) revealed that the Nsa2-N (1-58) construct has lost association with early and intermediate biogenesis factors—like Nop7, Erb1, Ytm1, Nog2, or Rsa4—but enriched late biogenesis factors such as Rei1 and Lsg1 that are known to be associated with cytoplasmic pre-60S particles [41,42]. This data suggests that Nsa2-N (1-58) somehow impairs the recycling of late 60S biogenesis factors. To test whether this biogenesis defect indeed occurs in the cytoplasm, we labeled the various Nsa2 mutants with GFP to determine their subcellular localization. However, this experiment did not reveal clear results, possibly due to a negative effect of the GFP tag fused to Nsa2 (J.B. data not shown). To circumvent this problem, we localized GFP-Nog1 upon overexpression of the various *nsa2* mutants. Here, we could observe that GFP-Nog1 was shifted from a predominant nuclear to a cytoplasmic location, when Nsa2-N (1-58) or Nsa2-N (1-84) were overexpressed (Figure 5c). The longer construct containing the RIS motif Nsa2-N (1-96) showed only a weak cytoplasmic signal for GFP-Nog1, but a few nuclear foci, in contrast to the Nsa2 N-terminal deletion constructs. Thus, overexpression of Nsa2 N-terminal fragments causes a block in recycling shuttling pre-60S biogenesis factors including Nog1 from the cytoplasm to the nucleus.

**Figure 6:**
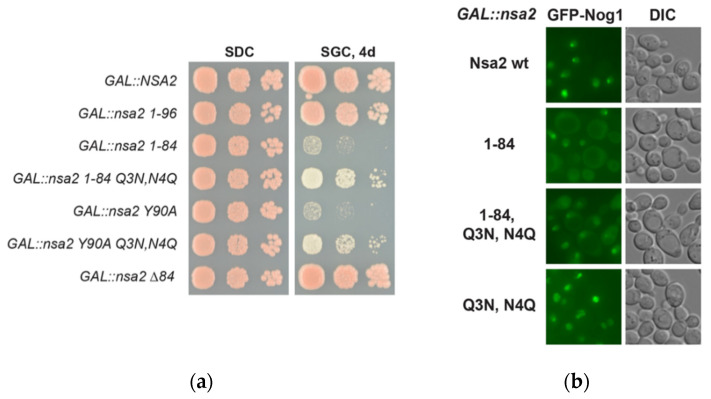

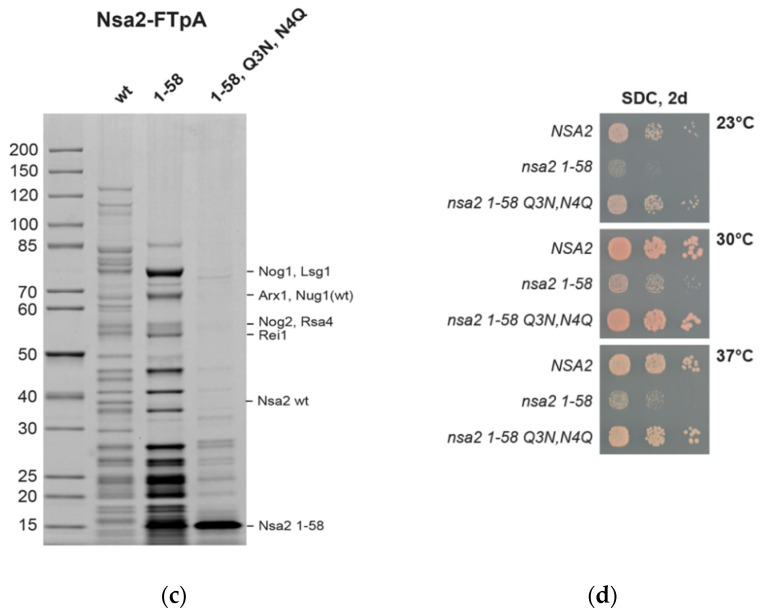
The Nsa2 Q3N,N4Q mutation attenuates the dominant-negative phenotype of the overproduced Nsa2 N-terminal domain. (**a**) Wild type yeast strain W303 carrying single-copy plasmid YCplac22 *NSA2*-L-HA with the indicated *nsa2* alleles under control of the *GAL1* promotor was spotted in a 1/10 dilution series on SDC-Trp (repression) and SGC-Trp (overexpression) plates and incubated at 30 °C for 3 (SDC) and 4 days (SGC), respectively. (**b**) Subcellular localization of GFP-Nog1 in yeast cells overexpressing the indicated *nsa2* alleles under *GAL1* promotor control (YCplac22 *GAL1::NSA2*-L-HA). (**c**) Tandem affinity-purification of Nsa2-FtpA constructs, inserted in YCplac111 *NSA2* L-FTpA plasmids. Final eluates were analyzed by 4–12% gradient SDS-PAGE and it was stained with Coomassie blue. (**d**) Growth phenotype of yeast strains used for the affinity-purification of the Nsa2 constructs shown in (**c**).

As we discovered that the Nsa2 N-terminus is essential for pre-60S targeting, whereas the lethal Nsa2 Q3N,N4Q was not defective for this function, we sought to better understand these different defects by engineering the Nsa2 Q3N,N4Q mutation also into the Nsa2 N-terminal constructs. Interestingly, the dominant-negative phenotype of *GAL::NSA2-N* (1-84) was partially rescued when the Q3N,N4Q mutation was additionally introduced (Figure 6a). Consistent with this observation, the cytoplasmic mis-localization of GFP-Nog1 observed upon Nsa2-N (1-84) overexpression was converted into a nuclear relocation when introducing the Nsa2 Q3N,N4Q mutation (Figure 6b). To correlate this subcellular location with biochemical data, we determined which 60S biogenesis factors are associated with these pre-60S particles. This revealed that the association of Nsa2-N (1-58) with pre-60S particles was largely lost when this same fragment in addition carried the Q3N,N4Q mutation (Figure 6c), which nicely explains the loss of the dominant-negative phenotype (Figure 6d). Taken together, the Nsa2 Q3N,N4Q mutation did not significantly affect binding to pre-ribosomal particles in the full-length protein, but has a major impact for binding when only N-terminal fragments were tested in the same way, underlining the importance of the Nsa2 N-terminal tip with its crucial Q3N residue. Thus, the Nsa2 Q3N,N4Q mutation is not sufficient to disturb pre-60S binding in the full-length protein, but it can do so when only Nsa2 N-terminal fragments were used. Thus, although these studies did not allow to fully decipher the molecular role of the Nsa2 Q3N,N4Q mutation, the data suggest that the Nsa2 N-terminal tip could affect the Nog1 GTPase, which may be required for a still unknown step in late pre-60S biogenesis (see Section 3).

## 3. Discussion

In this study, we showed that the Nsa2 N-terminus is required for targeting Nsa2 to pre-60S particles. Moreover, varying the linker length between the Rsa4 interacting sequence (RIS) and the Nas2 N-terminus further disturbs the pre-60S assembly pathway. Likewise, the conservative mutation Nsa2 Q3N leads to a lethal phenotype. Together with the recent insights into the various pre-60S structures, our findings shine light on the complex role of Nsa2 in pre-60S assembly.

Previously, it has been found that the release of Nsa2 is important for the maturation of the peptidyl transferase center (PTC), since Nsa2 keeps the rRNA helix H89 in a premature orientation [16,36]. It has been suggested that a mechanical force generated by the Rea1 ATPase is transmitted via Rsa4 to Nsa2, which initiates the rearrangement of the nascent PTC [16]. However, more recent structural data suggested that the Nsa2 conformation stays unaltered in the pre-60S ribosomal particles after the release of Rsa4. This indicates that not only the power stroke by the Rea1 ATPase, but also other subsequent event(s) could play a major role in the release of Nsa2. Nevertheless, the N-terminus of Nsa2 loses its dominant-negative phenotype, when the RIS is included, supporting an important functional connection of the Nsa2-N domain to the Rea1-mediated Rsa4 release.

Interestingly, the essential Nsa2 Q3 residue points to the GTP binding pocket of Nog1, which appears to carry GTP in nuclear pre-60S particles [36]. Previously, it was observed that the G223A mutation within the Nog1 GTPase domain causes a dominant-negative phenotype and accumulates in cytoplasmic pre-60S particles [24,43]. Therefore, it appears that GTP hydrolysis is required for the (cytoplasmic) release of Nog1. Since the shortening of Nsa2 Q3 by only one CH_2_ group (Q3N mutation) already leads to strong 60S assembly defects, we suggest that this Nsa2–Nog1 contact is of mechanistic relevance for the pre-60S maturation pathway. Initially, we hypothesized that Nsa2-N could act as a GAP-like activator of the Nog1 GTPase. However, if GTP hydrolysis by Nog1 requires Nsa2, one would expect that the mutation of the Nsa2 Q3 residue should cause a dominant-negative phenotype and tentatively an accumulation of Nsa2 Q3N and Nog1 on cytoplasmic pre-60S particles, which however could not be observed. Alternatively, Nsa2 Q3 might be a negative regulator of Nog1′s GTPase activity. In such a scenario, premature GTP hydrolysis due to the Nsa2 Q3N mutation might cause to an untimely release of Nog1. However, purification of the Nsa2 Q3N mutant still showed normal amounts of Nog1. Yet, this might be explained by only a partial release, in which the Nsa2 N-terminus with the attached Nog1 GTPase domain are being released, whereas the Nog1 C-terminus remains still attached to pre-60S particles via its insertion into the nascent exit tunnel [36]. Such an intermediate was recently detected by cryo-EM [22]. Thus, the complete Nog1 release depends on its GTPase function and the ATPase Drg1, which acts on Rlp24, an intimate binding partner of the Nog1 C-terminus [24,25,36]. Accordingly, late pre-60S particles purified from the *nog1 G223A* mutant or a *drg1* mutant show an enrichment of Nog1, but only a co-enrichment of Nsa2 is seen in the *nog1* mutant [24]. This indicates that the release of Nsa2 is linked to the (partial) release of the Nog1 N-terminus and GTPase domain. These observations suggest that the Nsa2 Q3 mutant might cause a premature release of the Nog1 N-domain and GTPase domain, which in turn destabilizes the Nsa2 binding to pre-60S particles. This could be an explanation, why the Nsa2 Q3 mutation does not exhibit a dominant-negative phenotype, but attenuates the dominant-negative phenotype of the Nsa2-N (1-58) or (1-84) overexpression constructs.

Accordingly, the functional interplay between Nsa2 and Nog1 during PTC formation might be more complex than previously envisioned. The N-terminus of Nsa2 is positioned in close proximity of the Sarcin–Ricin-loop (SRL) of the 25S rRNA. Recent cryo-EM analysis showed a subtle change of A3027 in the SRL in the presence and absence of Nsa2 [6,22]. Thus, this rRNA could be locally involved in activation of the Nog1 GTPase. Another putative regulator could be the Nog2 GTPase that contacts the Nog1-N and GTPase domain via its N-terminus (residues 129–182). Interestingly, the release of Nog2 is a trigger for the recruitment of the export adaptor Nmd3 [18]. Moreover, the Nsa2 N-terminus acts as a placeholder for Rpl40 (eL40), whose binding seems to correlate with the release of the Nog1 GTPase. However, also Yvh1 is strategically positioned to potentially influence Nog1’s GTPase activity. Finally, also a yet unidentified factor that only transiently binds after Nsa2 release could be the critical factor that triggers Nog1 release allowing the rRNA relocation for PTC maturation.

In summary, our molecular characterization of Nsa2 contributes to a more detailed understanding of Nsa2´s role in the complicated maturation of the PTC. Due to the extremely conserved amino acid sequence of Nsa2, we propose that our findings can be transferred to other eukaryotic organisms including human cells. Here, our data will contribute to the understanding of TINP1 function, which is functionally connected to the proliferation of cancer cells [33].

## 4. Materials and Methods

Plasmids and yeast strains used in this study are listed in Supplementary Materials Tables S2 and S3, respectively, including previously published material [16,42,44].

Cultivation and transformation of yeast and bacteria was done according to standard procedures.

Yeast integration were done as previously described [45]. Sedimentation analysis of cell lysates using a sucrose gradient has been specified before [46]. Antibodies used for western blot analysis have been obtained from the following sources: anti-Rpl35 [47], anti-Nop7 [24], anti Rei1 [48], anti-Rsa4 [49], anti-Nog1 [50], anti Nog2 [50], anti Nmd3 [21], anti-HA from rat (Merck, Darmstadt, Germany, 12013819001 Roche), and anti-proteinA (Merck, Darmstadt, Germany, P1291 Sigma-Aldrich). Semi-quantitative mass spectroscopy was analyzed using the MaxQuant software [51], protein alignment was done with the Clustal Omega [37] and Jalview [38] software, structural inspection and figure preparation of pre-60S particles was done using Chimera [39].

### 4.1. Affinity Purification

Two-step purification using the FTpA-tag (Flag-TEV-2x proteinA) was done according to previous protocols [52]. Please note, functional C-terminal tagging of Nsa2 requires a linker sequence (ASSYTAPQPGLGGS) to allow a successful purification, whereas N-terminal tagging is not functional [16] (H.P., J.B. unpublished data). The purification of Nsa2 mutants was done in a *NSA2* wild type depleted background (Figure 2c). For this purpose a *nsa2∆* strain was complemented by plasmid Ycplac22 *GAL1:*:*NSA2*-L-HA, were the *NSA2* is under control of the inducible *GAL1* promotor (*GAL::NSA2*). The mutants were expressed from plasmids pMT Leu2 NSA2-L-FtpA under the endogenous *NSA2* promotor. Cells were cultured in 2l SGC-Leu-Trp (galactose) before cells were centrifuged and resuspended in 2 l SDC-Leu-Trp (glucose) and incubated for additional 6 h before harvesting for purification.

### 4.2. Live Cell Imaging

Fluorescence microscopy was done as described in [53] using an Imager Z1 (Carl Zeiss, Oberkochen, Germany) equipped with a 100x NA 1.4 Plan-Apo-Chromat Oil immersion lens (Carl Zeiss) and DICIII and HE-EGFP, filter set, respectively. Images were taken by an AxioCamMRm camera (Carl Zeiss) controlled by software AxioVision 4.9.1 (Carl Zeiss) at resolution 1388 × 1040 (Binning 1 × 1, gain factor 1). GFP-Nog1 cells (see Supplementary Materials, Table S3) carrying mutants NSA2 mutants under control of the *GAL1* promotor (Ycplac22 *GAL1*::*NSA2*-HA or Yeplac112 *GAL1::NSA2*-L-Flag) were grown in SRC-Leu-Ade (Plasmid pASZ11 ADE2 was transformed in the yeast cells to suppress auto-fluorescence background) before shift for 6 h to SGC-Leu-Ade and subsequent lice cell imaging.

## Supplementary Materials

Supplementary materials can be found at https://www.mdpi.com/1422-0067/21/23/9108/s1.

## Abbreviations

ptA	ProteinA
FtpA	Flag-TEV-proteinA
RIS	Rsa4 interacting sequence
LD	linear dichroism
PTC	Peptidyl tranferase center
SRL	Sarcin–Ricin-loop
