# Supplementary material for: Mutational Analysis of the Nsa2 N-Terminus Reveals Its Essential Role in Ribosomal 60S Subunit Assembly
Source: Int J Mol Sci. 2020 Nov 30;21(23):9108. doi: 10.3390/ijms21239108 (PMC7730687; doi:10.3390/ijms21239108)
Supplement: Supplementary file 1 [file ijms-21-09108-s001.zip › Table S1 , S2, S3 - Mutants, Plasmids,Yeast strains.docx]

**Supplementary Table S1.** Nsa2 mutants analyzed in this study

| **Name** | **Mutation** | **Viability** | **Dom. Negative** | **Pre-60S Associated** |
| --- | --- | --- | --- | --- |
| Nsa2 ∆4 | Deletion of aa 1-4 | Lethal | None | Yes |
| Nsa2 ∆6 | Deletion of aa 1-6 | Lethal | None | Yes |
| Nsa2 Q3A,N4A | Q3🡪A, N4🡪A | Lethal | None | Yes |
| Nsa2 Q3N,N4Q | Q3🡪N, N4🡪Q | Lethal | None | Yes |
| Nsa2 Q3N | Q3🡪N | Lethal | None | Yes |
| Nsa2 N4Q | N4🡪Q | Viable | None | Yes |
| Nsa2 ∆14 | Deletion of aa 1-14 | Lethal | Weak | Yes |
| Nsa2 ∆34 | Deletion of aa 1-34 | Lethal | None | Slightly reduced |
| Nsa2 ∆58 | Deletion of aa 1-58 | Lethal | None | reduced |
| Nsa2 ∆84 | Deletion of aa 1-84 | Lethal | None | No |
| Nsa2 1-14 | Deletion of aa 15-261 | Lethal | None | No tested |
| Nsa2 1-34 | Deletion of aa 35-261 | Lethal | Strong | reduced |
| Nsa2 1-58 | Deletion of aa 59-261 | Lethal | Strong | Yes, cytoplasmic |
| Nsa2 1-84 | Deletion of aa 85-261 | Lethal | Strong | Yes, cytoplasmic |
| Nsa2 1-96 | Deletion of aa 97-261 | Lethal | None | Yes |
| Nsa2 L0 | Deletion of (S_77_KPLDTD_83_) | Lethal | Strong | No tested |
| Nsa2 L2 | (S_77_KPLDTD_83_) replaced by GS | Lethal | Strong | No tested |
| Nsa2 L4 | (S_77_KPLDTD_83_) replaced by GSGS | Viable | None | No tested |
| Nsa2 L6 | (S_77_KPLDTD_83_) replaced by GSGSGS | Viable | None | No tested |
| Nsa2 L7 | (S_77_KPLDTD_83_) replaced by GSGSGSG | Viable | None | No tested |
| Nsa2 L8 | (S_77_KPLDTD_83_) replaced by GSGSGSGS | Viable | None | No tested |
| Nsa2 L10 | (S_77_KPLDTD_83_) replaced by GSGSGSGSGS | Viable | None | No tested |
| Nsa2 L12 | (S_77_KPLDTD_83_) replaced by GSGSGSGSGGS | Lethal | Strong | No tested |
| Nsa2 1-58, Q3N,N4Q | Deletion of aa 59-261, Q3🡪N, N4🡪Q | Lethal | Weak | No |
| Nsa2 1-84, Q3N,N4Q | Deletion of aa 85-261, Q3🡪N, N4🡪Q | Lethal | Weak | Not tested |

**Supplementary Table S2.** Plasmids used in this study

| **Name** | **Number** | **Genotype** | **Reference** |
| --- | --- | --- | --- |
| YCplac22 NSA2-L-HA | 5931 | *TRP1*, *ARS*/*CEN*, *Amp*, *NSA2* Promotor, *NSA2-*Linker-HA, Linker before tag: ASSYTAPQPGLGGS | This study |
| YCplac22 *nsa2* ∆14-L-HA | 5932 | dto, Nsa2 15-261aa | This study |
| YCplac22 *nsa2* ∆34-L-HA | 5933 | dto, Nsa2 35-261aa | This study |
| YCplac22 *nsa2* ∆58-L-HA | 5934 | dto, Nsa2 59-261aa | This study |
| YCplac22 *nsa2* ∆84-L-HA | 5935 | dto, Nsa2 85-261aa | This study |
| YCplac22 *nsa2* 1-14-HA | 5936 | dto, Nsa2 1-14 aa, No linker before tag | This study |
| YCplac22 *nsa2* 1-34-HA | 5937 | dto, Nsa2 1-34 aa, No linker before tag | This study |
| YCplac22 *nsa2* 1-58-HA | 5938 | dto, Nsa2 1-58 aa, No linker before tag | This study |
| YCplac22 *nsa2* 1-84-HA | 5939 | dto, Nsa2 1-84 aa, No linker before tag | This study |
| YCplac22 *nsa2* 1-96-HA | 5940 | dto, Nsa2 1-96 aa, No linker before tag | This study |
| YCplac22 NSA2 | 5911 | *TRP1*, *ARS/CEN*, *Amp*, *NSA2* Promotor, *NSA2, no tag* | This study |
| YCplac22 *nsa2* L0 | 5956 | dto, Internal linker (S_77_KPLDTD_83_) has been deleted | This study |
| YCplac22 *nsa2* L2 | 5957 | dto, Internal linker (S_77_KPLDTD_83_) has been replaced by GS | This study |
| YCplac22 *nsa2* L4 | 5958 | dto, Internal linker (S_77_KPLDTD_83_) has been replaced by GSGS | This study |
| YCplac22 *nsa2* L6 | 5959 | dto, Internal linker (S_77_KPLDTD_83_) has been replaced by GSGSGS | This study |
| YCplac22 *nsa2* L7 | 5960 | dto, Internal linker (S_77_KPLDTD_83_) has been replaced by GSGSGSG | This study |
| YCplac22 *nsa2* L8 | 5961 | dto, Internal linker (S_77_KPLDTD_83_) has been replaced by GSGSGSGS | This study |
| YCplac22 *nsa2* L10 | 5962 | dto, Internal linker (S_77_KPLDTD_83_) has been replaced by GSGSGSGSGS | This study |
| YCplac22 *nsa2* L12 | 5963 | dto, Internal linker (S_77_KPLDTD_83_) has been replaced by GSGSGSGSGSGS | This study |
| YCplac22 *GAL1*::*NSA2*-L-HA | 5946 | TRP1, ARS/CEN, Amp, *GAL1* promotor, *NSA2*-Linker-HA Linker: ASSYTAPQPGLGGS | This study |
| YCplac22 *GAL1*::*nsa2* ∆14-L-HA |  | dto, Nsa2 15-261aa | This study |
| YCplac22 *GAL1*::*nsa2* ∆34-L-HA | 5948 | dto, Nsa2 35-261aa | This study |
| YCplac22 *GAL1*::*nsa2* ∆58-L-HA | 5949 | dto, Nsa2 59-261aa | This study |
| YCplac22 *GAL1*::*nsa2* ∆84-L-HA | 5950 | dto, Nsa2 85-261aa | This study |
| YCplac22 *GAL1*::*nsa2* 1-14-HA | 5951 | dto, Nsa2 1-14 aa, No linker | This study |
| YCplac22 *GAL1*::*nsa2* 1-34-HA | 5952 | dto, Nsa2 1-34 aa, No linker | This study |
| YCplac22 *GAL1*::*nsa2* 1-58-HA | 5953 | dto, Nsa2 1-58 aa, No linker | This study |
| YCplac22 *GAL1*::*nsa2* 1-84-HA | 5954 | dto, Nsa2 1-84 aa, No linker | This study |
| YCplac22 *GAL1*::*nsa2* 1-96-HA | 5955 | dto, Nsa2 1-96 aa, No linker | This study |
| YCplac22 *GAL1*::*nsa2* 1-84 Q3N,N4Q HA | 6695 | dto, Nsa2 1-84 aa, Q3N, N4Q, No linker | This study |
| YCplac22 *GAL1*::*nsa2* Y90A L-HA | 6693 | dto, Nsa2 1-261 aa, Y90A | This study |
| YCplac22 *GAL1*::*nsa2* Q3N,N4Q Y90A L-HA | 6694 | dto, Nsa2 1-261 aa, Q3N, N4Q, Y90A | This study |
| YEplac112 *GAL1*::NSA2 L-Flag | 5976 | 2µ, *TRP1, Amp, GAL1* Promotor, Flag-tag, Linker between *NSA2* and tag: ASSYTAPQPGLGGS | [16] |
| YEplac112 *GAL1*::*nsa2* Y90A L-Flag | 5977 | dto, Y90A | [16] |
| YEplac112 *GAL1*::*nsa2* ∆34 L-Flag | 5979 | dto, Nsa2 35-261aa | This study |
| YEplac112 *GAL1*::*nsa2* L0 L-Flag | 5986 | dto, Internal linker (S_77_KPLDTD_83_) has been deleted | This study |
| YEplac112 *GAL1*::*nsa2* L2 L-Flag |  | dto, Internal linker (S_77_KPLDTD_83_) has been replaced by GS | This study |
| YEplac112 *GAL1*::*nsa2* L12 L-Flag | 5987 | dto, Internal linker (S_77_KPLDTD_83_) has been replaced by GSGSGSGSGSGS | This study |
| YEplac112 *GAL1*::*nsa2* 1-84-Flag | 5983 | 2µ, TRP1, Amp, *GAL1* Promotor, Flag-tag, no linker between Nsa2 fragment and tag | This study |
| YEplac112 *GAL1*::*nsa2* 1-96-Flag | 5984 | 2µ, TRP1, Amp, *GAL1* Promotor, Flag-tag, no linker between Nsa2 fragment and tag | This study |
| YCplac111 *NSA2 L-FTpA* | 5423 | *LEU2, ARS/CEN, AmpR, NSA2* Promotor, *NSA2*-LINKER-FLAG-TEV-pA, Linker before tag: ASSYTAPQPGLGGS | [16] |
| YCplac111 *nsa2 ∆34 L-FTpA* | 5898 | dto, Nsa2 35-261aa | This study |
| YCplac111 *nsa2 ∆58 L-FTpA* | 5899 | dto, Nsa2 59-261aa | This study |
| YCplac111 *nsa2 ∆84 L-FTpA* | 5900 | dto, Nsa2 85-261aa | This study |
| YCplac111 *nsa2 1-34 -FTpA* | 5972 | dto, Nsa2 1-34 aa, No linker | This study |
| YCplac111 *nsa2 1-58 -FTpA* | 5973 | dto, Nsa2 1-58 aa, No linker | This study |
| YCplac111 *nsa2 1-84 -FTpA* | 5974 | dto, Nsa2 1-84 aa, No linker | This study |
| YCplac111 *nsa2 1-96 -FTpA* | 5975 | dto, Nsa2 1-96 aa, No linker | This study |
| pMT_Leu2 *NSA2* | 6660 | 2µ, *LEU2, Amp*, *NSA2* Promotor, *NSA2* | This study |
| pMT_Leu2 *nsa2 ∆4* | 6661 | dto *nsa2 (5-261aa)* | This study |
| pMT_Leu2 *nsa2 ∆6* | 6662 | dto *nsa2 (7-261aa)* | This study |
| pMT_Leu2 *nsa2 Q3A,N4A* | 6663 | dto *nsa2 Q3A, N4A* | This study |
| pMT_Leu2 *nsa2 Q3N,N4Q* | 6664 | dto *nsa2 Q3N, N4Q* | This study |
| pMT_Leu2 *nsa2 Q3N* | 6665 | dto *nsa2 Q3N* | This study |
| pMT_Leu2 *nsa2 N4Q* | 6666 | dto *nsa2 N4Q* | This study |
| pMT_Leu2 *NSA2-L-FTpA* | 6667 | 2µ, *LEU2, Amp*, *NSA2* Promotor, *NSA2* LINKER-FLAG-TEV-pA, Linker before tag: ASSYTAPQPGLGGS | This study |
| pMT_Leu2 *nsa2 ∆4-L-FTpA* | 6669 | dto *nsa2 (5-261aa)* | This study |
| pMT_Leu2 *nsa2 Q3A,N4A-L-FTpA* | 6671 | dto *nsa2 Q3A, N4A* | This study |
| pMT_Leu2 *nsa2 Q3N,N4Q-L-FTpA* | 6672 | dto *nsa2 Q3N, N4Q* | This study |
| pMT_Leu2 *nsa2 Q3N-L-FTpA* | 6673 | dto *nsa2 Q3N* | This study |
| pMT_Leu2 *nsa2 N4Q-L-FTpA* | 6666 | dto *nsa2 N4Q* | This study |
| pMT_Leu2 *GAL1*::*NSA2* | 6653 | 2µ, *LEU2, Amp*, *GAL1* Promotor, *NSA2* | This study |
| pMT_Leu2 *GAL1*::*nsa2 ∆4* | 6654 | dto *nsa2 (5-261aa)* | This study |
| pMT_Leu2 *GAL1*::*nsa2 ∆6* | 6655 | dto *nsa2 (7-261aa)* | This study |
| pMT_Leu2 *GAL1*::*nsa2 Q3A,N4A* | 6656 | dto *nsa2 Q3A, N4A* | This study |
| pMT_Leu2 *GAL1*::*nsa2 Q3N,N4Q* | 6657 | dto *nsa2 Q3N, N4Q* | This study |

**Supplementary Table S3.** Yeast strains used in this study

| **Name** | **Number** | **Genotype** | **Reference** |
| --- | --- | --- | --- |
| Nsa2∆ | Y4267 | *nsa2::kanMX6, ade2-1, ura3-1, his3-11.15, leu2-3.112, trp1-1, can1-100,* MATa*,* pRS316*-NSA2* | [16] |
| DS1-2b | Y2197 | *his3-∆200 leu2-∆1, trp1-∆63 ura3-52,* MATα | [42] |
| W303 | Y1721 | *ade2-1, his3-11,15, leu2-3,112, trp1-1, ura3-1, can1-100,* MATa | [44] |
| Arx1-FTpA, Nsa2∆ | Y5549 | *ARX1*-*FTpA*::*natNT2*, *nsa2*::*kanMX6*, *ade2-1*, *ura3-1*, *his3-11.15*, *leu2-3.112*, *trp1-1*, *can1-100*, MATa | [16] |
| Nog1-GFP, Nsa2∆ | Y6161 | *ade2-1*, *ura3-10*, *his3-11.15*, *leu2-3.112*, *trp1-1*, *can1-100*, *nsa2*::*kanMX6*, *P_RSA4_* GFP-*NOG1*::*natNT2,* MATa | This study |
| Nmd3-HTpA  Nsa2-L-Flag | Y6287 | *ade2-1*, *ura3-10*, *his3-11.15*, *leu2-3.112*, *trp1-1*, *can1-100*, *NSA2-L-Flag*::*natNT2*, *NMD3-HTpA::HIS3 (*HIS_6_-TEV-proteinA*)* | This study |
