# Supplementary figures and images for: Mutational Analysis of the Nsa2 N-Terminus Reveals Its Essential Role in Ribosomal 60S Subunit Assembly
Source: Int J Mol Sci. 2020 Nov 30;21(23):9108. doi: 10.3390/ijms21239108 (PMC7730687; doi:10.3390/ijms21239108)

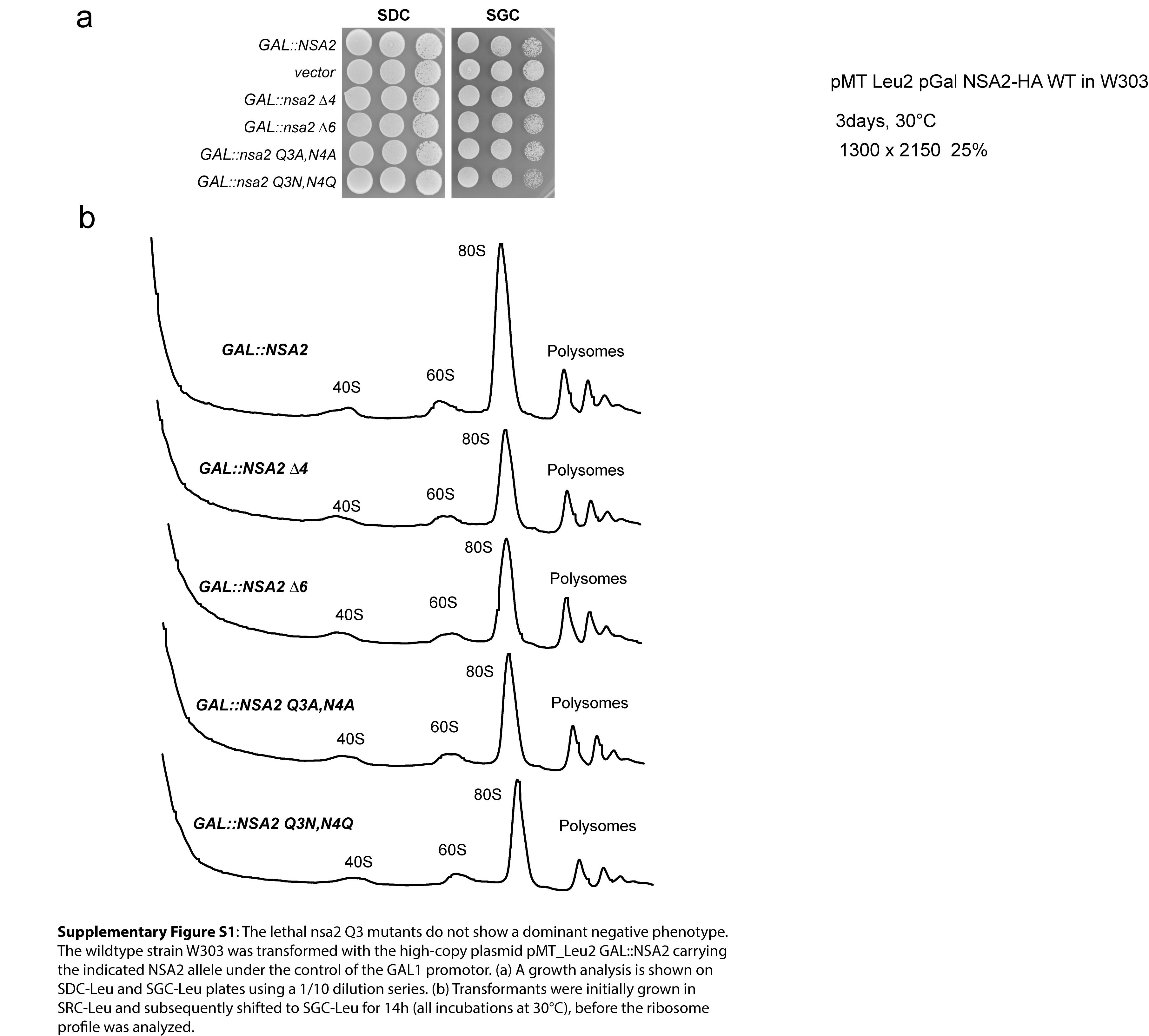

Supplement: Supplementary file 1 [file ijms-21-09108-s001.zip › Figure S1.tif]
